# A method to quantify the reduction of back and hip muscle fatigue of lift-support exoskeletons

**DOI:** 10.1017/wtc.2022.32

**Published:** 2023-01-23

**Authors:** Rachel M. van Sluijs, David Rodriguez-Cianca, Clara B. Sanz-Morère, Stefano Massardi, Volker Bartenbach, Diego Torricelli

**Affiliations:** 1 Auxivo AG, Zurich, Switzerland; 2 Cajal Institute, Spanish National Research Council, Madrid, Spain; 3 Center for Clinical Neuroscience – Hospital Los Madroños, Madrid, Spain

**Keywords:** biomechanics, exoskeletons, exosuits, industry

## Abstract

Cumulative back muscle fatigue plays a role in the occurrence of low-back injuries in occupations that require repetitive lifting of heavy loads and working in forward leaning postures. Lift-support exoskeletons have the potential to reduce back and hip muscle activity, thereby delaying the onset of fatigue in these muscles. Therefore, exoskeletons are being considered a potentially important tool to further reduce workload-related injuries. However, today no standards have been established on how to benchmark the support level of lift-support exoskeletons. This work proposes an experimental protocol to quantify the support level of a lift-support exoskeletons on instant changes in muscle activity and fatigue development while maintaining a static forward leaning posture. It then applies the protocol to experimentally assess the effect of the support provided by a commercially available lift-support exoskeleton, the LiftSuit 2.0 (Auxivo AG, Schwerzenbach, Switzerland), on the user. In a sample of 14 participants, the amplitude of the muscle activity of the back muscles 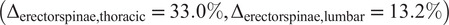 and hip muscles ( 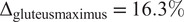 ) was significantly reduced. Wearing the exoskeleton significantly reduced the amount of fatigue developed during the task ( 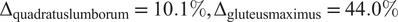 ). Changes in muscle fatigue can be objectively recorded and correlated with relevant changes for exoskeleton users: the time a task can be performed and perceived low-back fatigue. Thus, including such measures of fatigue in standardized benchmarking procedures will help quantify the benefits of exoskeletons for occupational use.

## 1. Introduction

Heavy physical work including repetitive lifting and forward leaning can lead to musculoskeletal disorders and low-back pain (Luttmann et al., 2003), with significant reduction of quality of life (Dueñas et al., 2016), temporary or permanent work incapacity (Baldwin, 2004), and large financial burden to society (Bevan, 2015). The US Labor Bureau reports that around 30% of days away from work are caused by musculoskeletal disorders (Bureau of Labor Statistics, 2016). This issue is common across many application domains (Bureau of Labor Statistics, 2016; Kok et al., 2019), including healthcare (Smedley et al., 1995), construction (Latza et al., 2002), fishing (Nørgaard Remmen et al., 2021), and agriculture (Holmberg et al., 2003). Considering all occupations, 41% of workers report backache complaints (Kok et al., 2019).

Wearable exoskeletons for industrial (or occupational) use are now gaining interest as a possible solution to provide support to workers and reduce the prevalence of long-term musculoskeletal disorders. Though several types of exoskeletons have been developed to support upper limbs and low-back during industrial applications (Crea et al., 2021; Pesenti et al., 2021; De Bock et al., 2022), most real-world and field studies have been carried out with commercial passive exoskeletons mainly because of their portability and affordability compared to active ones. Passive exoskeletons have been proven useful in automation (Hensel and Keil, 2019; Pacifico et al., 2020), healthcare (Settembre et al., 2020), and industry (Pacifico et al., 2022) environments, with a positive effect in biomechanical parameters (i.e., reduced lumbar compression), metabolic cost, and functional domains but, above all, in muscle activation reduction (de Looze et al., 2016; Bär et al., 2021; Pesenti et al., 2021).

Most of those studies focused on assessing short-term changes in muscle activation (De Bock et al., 2022). However, it has been shown that low-back injuries are also caused by cumulative muscle fatigue (Garg and Moore, 1992). Muscle fatigue can be defined as a reduction in the force-generating capacity of muscles during prolonged use, which is accompanied by an increased perceived effort and might lead to the inability of performing a task (Barry and Enoka, 2007). Thus, the occurrence of muscle fatigue is related to negative effects on task performance and productivity. Low-back muscle fatigue develops both during dynamic lifting tasks and prolonged exposures to static bending positions (Potvin and Norman, 1993; Bonato et al., 2003), such as those commonly performed by workers in the automotive industry (Hensel and Keil, 2019. Despite this evident relationship between fatigue and injury risk, so far only a few studies have assessed the effects of lift-support exoskeletons on the development of muscle fatigue during dynamic lifting (Lotz et al., 2009; Poon et al., 2019; Yin et al., 2019) and forward leaning (Bosch et al., 2016; Lamers et al., 2020). The reason why a reduction in rate of fatigue is rarely used to benchmark lift exoskeletons could be that measuring fatigue in torso muscles is more challenging than in other body parts. In the extremities, the relationship between muscle activity and resulting forces can be measured objectively by using electrical stimulation of the innervating nerves (Garcia et al., 2016; Place and Millet, 2020). However, in the torso muscles, symptoms of fatigue (including changes in muscle activity) can only be quantified by means of voluntary force output (Davidson et al., 2004), surface electromyography (EMG) (studying the increased EMG amplitude or reduction in median frequency (MDF); Farina et al., 2003), and heart rate and endurance (time to discomfort or time to task failure; Bosch et al., 2016). Changes in MDF of the back and hip muscles are correlated with task endurance time (Coorevits et al., 2008) and subjective experience of lumbar muscle fatigue (Dedering et al., 1999), which are important factors for occupational exoskeleton users.

Nonetheless, some literature exists that quantifies the effect of exoskeletons on changes in fatigue during lifting. In Bosch et al. (2016), endurance was measured as the time passed until the participant felt discomfort, but no quantification of fatigue-related changes in EMG was performed. In Lamers et al. (2020), the authors report the effects on lumbar muscle fatigue of an elastic low-back exosuit in six participants by assessing the changes in the slope of the median frequencies of the muscle activity. Authors showed consistent reductions in fatigue rate of the lumbar muscles ranging from 26% to 87% in five of the six participants. Moreover, Lamers et al. used the reduction of the slope of the muscle median frequencies as a fatigue indicator, which has been shown to be consistent with self-reports of fatigue (Bonato et al., 2003) and to be linearly correlated with the accumulation of muscle metabolites implicated in the development of muscle fatigue during isometric contractions of the back extensor muscles (Mannion and Dolan, 1994).

So far, since literature regarding fatigue during lifting is not extensive, no protocol or method has yet been established as a standard to provide reliable, practical, and comparable fatigue-related performance indicators for exoskeletons (Crea et al., 2021; De Bock et al., 2022. Such standardized performance indicators are crucial for the wider adoption of exoskeletons, because they allow potential users to assess the suitability of an exoskeleton device for their specific use-case and compare different devices according to their requirements (Torricelli et al., 2020. Our research, inspired by the work of Lamers et al., aims to develop a reproducible, practical, and standardized protocol for measuring fatigue that can be used to benchmark and compare a wide range of lift- and back-support exoskeleton. Such a protocol needs (1) to include the assessment of the most relevant performance indicators, (2) to include tasks that are relevant for industrial applications, and at the same time it needs (3) to be reproducible in a practical way with an acceptable investment of resources including number of participants, time, and equipment to allow its wider adoption in the field. In the current work, we propose a protocol to induce and measure back muscle fatigue during forward leaning while holding an external load tailored to the user’s body weight and fitness and test the protocol using the commercially available Auxivo LiftSuit v2.0 (Auxivo AG, Schwerzenbach, Switzerland).

## 2. Methods

The work was conducted in the context of the H2020 EUROBENCH project, which aims to create a unified benchmarking framework for, among others, wearable robotic systems including exoskeletons to allow companies and/or researchers to test and compare the performance of their devices at any stage of development (https://eurobench2020.eu/). Measurements were conducted at the wearable robots testing facility in Hospital Los Madroños, Madrid, Spain. The study protocol (091/2021) was approved by the Spanish Research Council (CSIC). Participants signed the informed consent and the measurements were conducted in line with the Declaration of Helsinki.

### 2.1. Participants

Data were collected from 14 healthy individuals (9 female) between 21 and 35 years old (mean: 25.3 years; SD: 4.1 years). Participants’ body height ranged from 1.57 to 1.87 m (mean: 1.70 m; SD: 0.1 m) and their body weight was between 53 and 140 kg (mean: 70.7 kg; SD: 23.3 kg). Participants were recruited from the workers and collaborators of Hospital Los Madroños.

### 2.2. Exoskeleton

**Figure 1.**
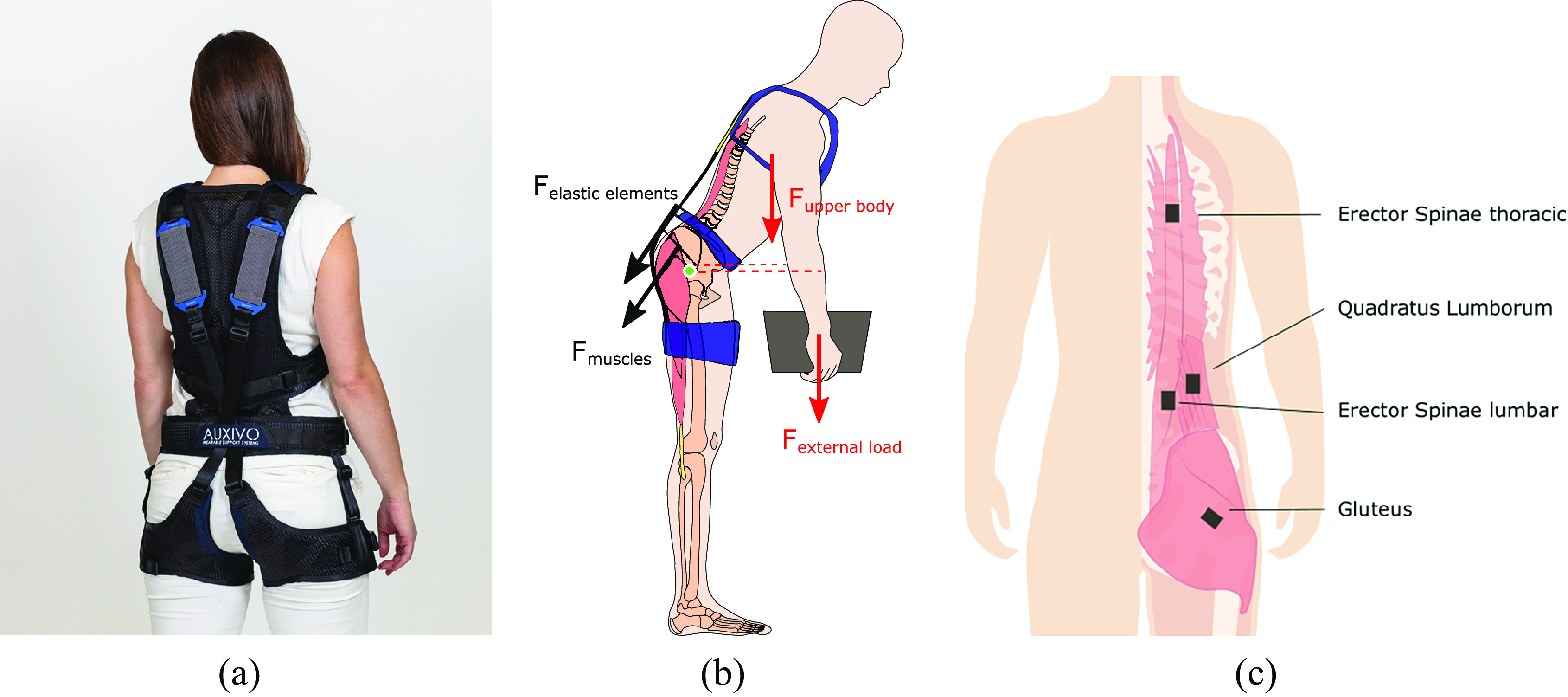
(a) The passive lift-support exoskeleton used in this study is the Auxivo LiftSuit v2.0. (b) During static forward leaning the load of the body, the external load, the muscles, and the exoskeleton create opposing moments around the center of rotation of the hip (in green). The moments generated by the weight of the upper body and the external load (in red) are counteracted by the muscles that create torque around the hip and the exoskeleton (in black). Dotted lines represent moment arms. (c) In this study, we measured the erector spinae at lumbar and thoracic level, the quadratus lumborum, and the gluteus maximus.

In this study, the LiftSuit v2.0 (Auxivo AG, Schwerzenbach, Switzerland) was used (Figure 1). The exoskeleton is available in two sizes: size S/M worn by 12 participants and size L/XL worn by two participants. The device includes two elastic bands located on the back of the user, which span between the torso and thighs. The bands are connected to the upper body via a vest and to each leg through a thigh cuff. A hip belt prevents the bands from slipping laterally off the back. The bands are stretched when the participant bends forward because the bending at the hip increases the distance between the vest and the cuffs along the hip and back. The stretched bands provide a force parallel to the human back and hip muscles.

The LiftSuit is activated by pulling two loops at the shoulder (see exoskeleton video manual provided in the Supplementary Material). When pulling the loops lightly, the bands on the back are shortened until the elastic bands lie flat against the vest. When pulling the loops further, the elastic bands can be pre-tensioned. This way, the user can regulate how much support is received. Participants were instructed to activate the LiftSuit, take the forward leaning position, and then adjust the activation until they subjectively perceived a comfortable and sufficient assistance to execute the task. The experimenter then corrected any asymmetry in band length to guarantee symmetric forces.

### 2.3. Experimental protocol

The study consisted of two measurements conducted on separate days. Visit 1 aimed to determine the level of the external load needed to induce detectable fatigue in the lumbar erector spinae. During visit 2, the effect of the exoskeleton on the development of fatigue was evaluated.

**Figure 2.**
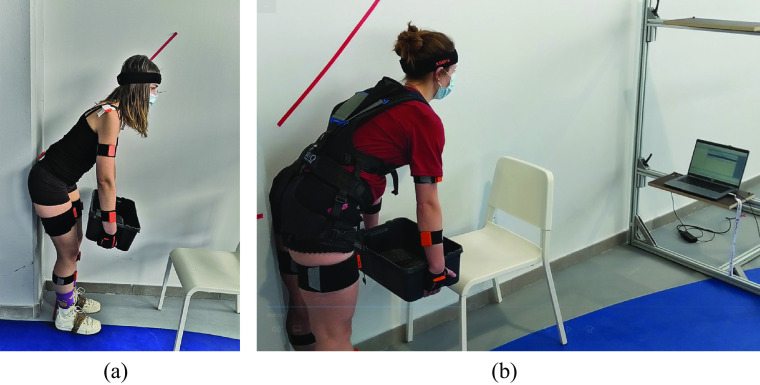
(a) Participants held a box of 20% body weight in a 45° forward leaning position. (b) Real-time feedback of trunk angle was provided on a screen in the line of sight of the participant.

During both visits, participants were instructed to hold a box while taking a forward leaning position. The angle of the upper body was 45° forward. The hip position was restrained to be maximally 10 cm behind the heels. During each trial, this position was held for 90 s. Real-time feedback of trunk angle was provided on a screen in the line of sight of the participant, see Figure 2. Participants were instructed to stay within a 10° window around 45° displayed as a line graph (*x*-axis: time (s); *y*-axis: angle (°) with axis limits of 40° to 50°), as well as a number visible inside the graph. Feedback was also introduced in other studies measuring static lifting for similar purposes (Potvin and Norman, 1993; Bonato et al., 2003). Looking at the screen promoted standing with an elongated spine and influenced participants head, neck, and shoulder position. No measures to control spine curvature were taken.

#### 2.3.1. Visit 1: Determination of external load

During visit 1, participants executed the task while holding a box weighing 20% of their body weight. We opted to customize the external load based on body weight, differently from Lamers et al. (2020), to try to induce a sufficient level of fatigue in all participants during the task. More precisely, Lamers et al. selected a fixed weight of 11 kg for all participants and did not observe signs of fatigue in 6 out of 12 participants. These six participants were subsequently excluded from their study. In order to ensure an adequate sample, we hypothesized that localized muscle fatigue could be induced in all participants by individualizing the external load to their body weight. Thus, we included one iteration to tune the external load to the participants’ fitness level. First, we measured the muscle activity of the lumbar erector spinae while leaning forward at 45  for 90 s. Fatigue develops gradually, but for the purpose of standardization, fatigue was considered to have appeared when the MDF was reduced by 10% (Potvin and Norman, 1993). If the MDF in the lumbar erector spinae dropped below this threshold during the task, the external load was considered suitable for the experiment. However, if the MDF reduction was less than 10%, the external load was increased to 25% of body weight for visit 2. If the participant experienced unacceptable discomfort or could not hold the load stable for 90 s, the load was reduced to 15% of body weight for visit 2. This new external load was not verified in another test cycle.

#### 2.3.2. Visit 2: Within person comparison

During visit 2, participants performed the same task, once with the exoskeleton and once without the exoskeleton, in randomized order. Between the two rounds, the participants had a break of at least 15 min.

### 2.4. Measures

The outcome measures of this study were muscle activation measured through surface EMG and kinematics using inertial measurement units (IMUs). The bilateral muscle activity of 4 back and hip muscles was measured: erector spinae at the level of the thorax, erector spinae at the lumbar level, quadratus lumborum, and gluteus maximus. Surface EMG electrodes were placed in accordance with the recommendation of Criswell (2010) on the following sites (Figure 1(c)): thoracic erector spinae (T-12 level approximately 2 cm laterally from the spine); lumbar erector spinae (L-3 level 2 cm laterally to the spine); quadratus lumborum (halfway between the 12th rib and the iliac crest, approximately 4 cm lateral to the erector spinae sensor); and gluteus maximus (half the distance between the trochanter (hip) and the sacral vertebrae in the middle of the muscle). These muscles were selected because they contribute to the hip moment during static forward leaning tasks (Elzanie and Borger, 2019), are expected or have been shown to fatigue during the task, and allow a comparison with existing literature. Specifically, all studies assessing fatigue measured the lumbar erector spinae including multifidus, longissimus and iliocostalis (Lotz et al., 2009; Poon et al., 2019; Yin et al., 2019; Lamers et al., 2020), and most the thoracic erector spinae (Lotz et al., 2009; Poon et al., 2019; Yin et al., 2019). Studies reporting on the difference between squat and stoop lifting focus on thoracic and lumbar erector spinae (Potvin and Norman, 1993; Wang et al., 2012) and abdominal muscles (Potvin and Norman, 1993). However, Lamers et al. (2020) recorded abdominal muscles and lattisimus dorsi but observed only low levels of abdominal muscle activity (<5%MVC) and no changes in MDF; therefore, the authors excluded these muscles from their fatigue analysis and the muscles are not included in the current protocol. Muscle activity was recorded with Trigno sensors using EMGworks Acquisition software (Delsys Ltd, Natick, United States). The system sampling frequency was 2048 Hz.

Full-body movement kinematics were captured using 17 IMU-based sensors MVN Awinda and MVN analyze/Animate software (Xsens Technologies B.V., Enschede, Netherlands). Though only 11 sensors are needed to build the anatomical model, the Xsens algorithms are only validated with full-body sensor configuration (17 IMUs). So, considering that the donning time of the full-body configuration was not notably higher than for the 11-IMU configuration, and in order to ensure the feedback reliability, we selected the full-body configuration. The signal was sampled at 60 Hz. A list of biometric parameters were measured for each participant and served as input for the software model: foot length, shoulder height, shoulder width, elbow span, wrist span, arm span, hip height, hip width, knee height, and ankle height. For the analysis, the start moment of the 90 s window the experimenter determined based on the participant finding a stable trunk angle.

In addition to the EMG and kinematics measurements, we quantified the mechanical support provided by the exoskeleton in order to evaluate how well the mechanical support translates into a physiological load reduction considering factors such as weight and size of the individual user. While the quantification of the mechanical support is of course exoskeleton specific, we consider it an important aspect as it shows how efficient an exoskeleton is in supporting its users. In case of the LiftSuit, the support during use can be quantified by measuring the stretch of the elastic elements of the exoskeleton with a measurement tape after the participant reached a stable position. To determine the relationship between stretch of the textile spring and the resulting force, a tensile test was performed according to European Standard (EN566). Because the two textile springs work in parallel, the force of the left and right textile spring was added up.

### 2.5. Data processing and statistical analysis

The EMG data were converted to .mat data files and processed and analyzed in MATLAB R2019b (MathWorks, Natick, United States). The signal was filtered with a 4th order Butterworth band-pass filter with cut-off frequencies of 10 and 500 Hz, and an infinite impulse response notch filter at 50 Hz with *Q* factor 20 to remove powerline noise. After visual inspection of the signal in the frequency domain and detection of noise potentially resulting from the proximity of the EMG and IMU sensors, two additional infinite impulse response notch filters were added (at 296 Hz and 370 Hz). Consecutively, the root mean square of the signal was calculated for each muscle for the entire duration of the task. The MDF of the signal was calculated every second. Linear regression analysis was used to quantify the change in MDF over time. In samples where the marginal effect was low (presence of little fatigue), the regression coefficient was unreliable. For this reason, regression lines with positive coefficient were treated as NaN (erector spinae thoracic = 3; quadratus lumborum = 2; gluteus maximus = 3). No marginal effects were observed in the lumbar erector spinae, since the protocol was tuned to induce fatigue in the lumbar erector spinae. Lamers and colleagues excluded participant wise based on marginal effects observed in the lumbar erector spinae (determined using regression analysis).

The key performance indicators in this study were (1) the change in muscle activity calculated as the RMS over the entire 90 s window and (2) the development of fatigue calculated as the slope of the regression line of the MDF. Pair-wise comparison of the two conditions (no exoskeleton (NoExo), exoskeleton (Exo)) was performed using paired sample t-tests for each performance indicator. The test outcomes were considered significant if p < .05.

## 3. Results

During visit 1, the initial external weight of 20% body weight led to a decrease in the MDF of the erector spinae lumbar of at least 10% within the 90 s time window for the majority of participants (57%). For 37% of participants, this MDF decrease threshold was not reached during visit 1 and the load was increased to 25% body weight for visit 2. The external loads used during visit 2 ranged between 11 and 21 kg: 15% of body weight for 1 participant, 20% of body weight for 8 participants, and 25% of body weight for 5 participants. During visit 2, the lumbar erector spinae MDF decrease was 24.4% in the NoExo condition (SD: 16.5%). A lumbar erector spinae MDF decrease below the defined 10% fatigue threshold was observed in two participants ( 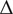 MDF = 8.16% and 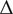 MDF = 8.07%) during the NoExo condition. All participants participated in the second visit and were included in the statistical analysis.

**Figure 3.**
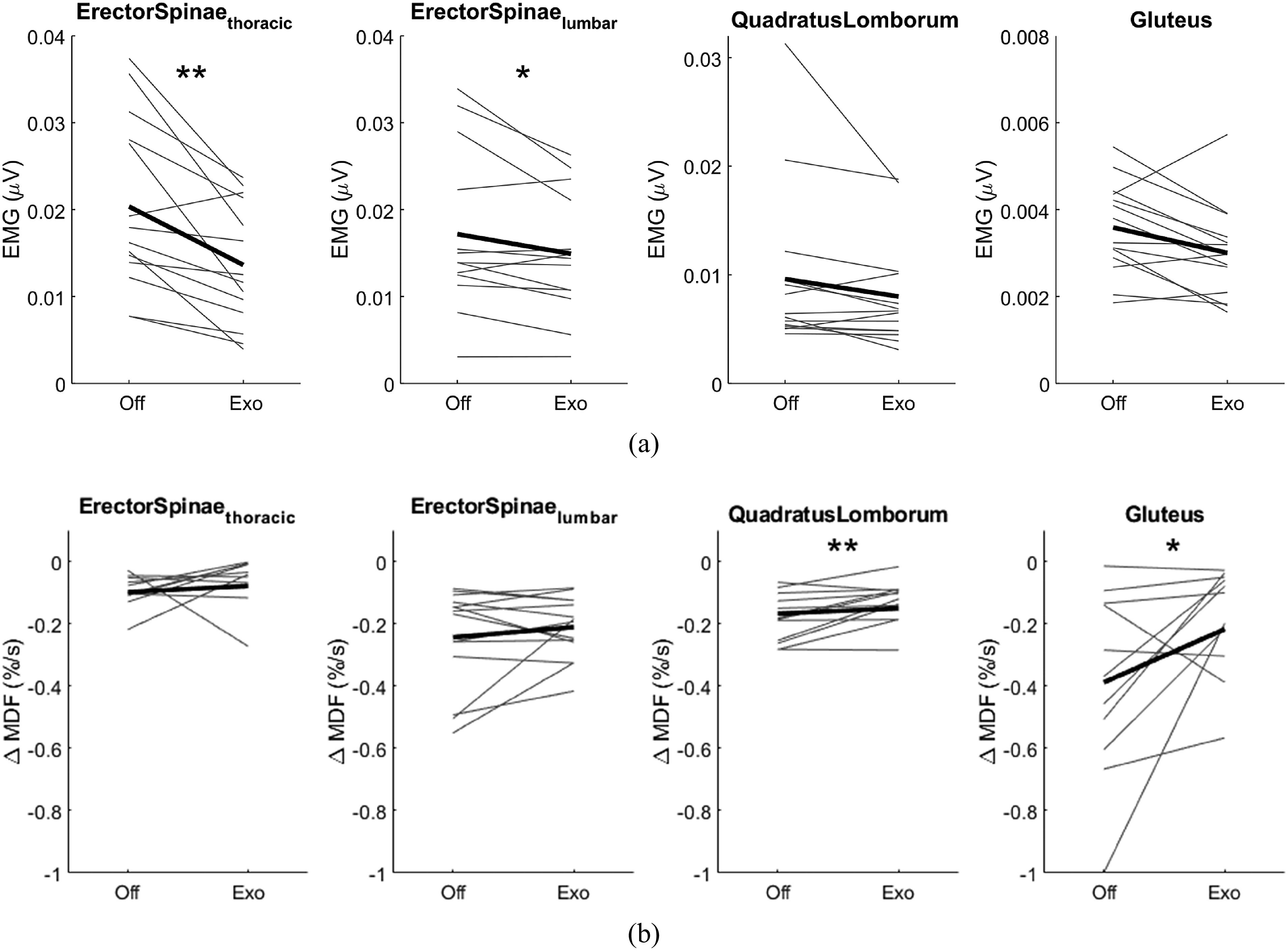
(a) Root mean square of the muscle activity of each muscle group. (b) Slope of the change in median frequency over time for each muscle group. Individual participants (grey lines), as well as the sample average (*n* = 14, black line), are plotted. Stars indicate statistically significant differences based on paired samples t-test analysis: *p < .05, **p < .01.

Significant reductions in muscle activity when wearing the exoskeleton were observed in the erector spinae in both the thoracic (*p* = .001) and lumbar region (*p* = .025), as well as in the gluteus maximus (*p* = .020), see Table 1. The largest effect of the exoskeleton was observed in the erector spinae at thoracic level (33.0% reduced compared to no exoskeleton), followed by the quadratus lumborum (16.7%) and gluteus maximus (16.3%), see Figure 3(a).

**Table 1.** Change in muscle activity when wearing the Exo with respect to the NoExo condition across the sample (*n* = 14)

	Change in activity (NoExo – Exo)			Paired t-test		
Muscle group	Mean ( 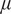 V)	SD ( 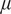 V)	Mean (%NoExo)	*t*	*p*-value	Cohen’s *d*
Erector spinae 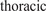	6.71E-03	6.21E-03	33.0	4.05	.001	1.08
Erector spinae 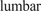	2.26E-03	3.34E-03	13.2	2.53	.025	0.68
Quadratus lumborum	1.61E-03	3.53E-03	16.7	1.70	.112	0.46
Gluteus maximus	5.85E-04	8.27E-04	16.3	2.65	.020	0.71

*Note.* Mean and SD of the absolute change ( 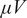 ) and change as % of NoExo condition, as well as the test statistic *t*, the *p*-value, and the effect size (Cohen’s *d*) of the paired samples *t*-test are reported.

**Figure 4.**
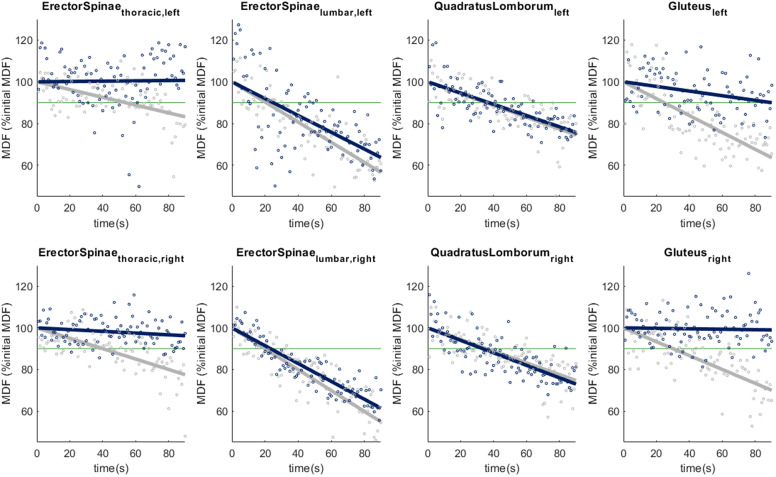
Change in median frequency (MDF) over time for one participant. The MDF of the EMG signal was calculated for non-overlapping 1 s time windows (dots). A linear regression line was calculated for each condition: NoExo (grey) and Exo (blue).

Figure 4 shows the change in MDF of the muscle activity over the entire period of the forward leaning task for one participant. The external load was adapted based on the results of visit 1 to induce a measurable change in MDF in the lumbar erector spinae. On an inter-subject average level, the slopes of all muscles were less steep when performing the task in the Exo condition compared to the NoExo condition, Figure 3(b). This flattening of the slope was 44.0% for the gluteus maximus, 20.1% for the erector spinae at the thoracic level, and 10.1% for the quadratus lumborum. The flatting of the slope of the gluteus maximus was larger (44.0% NoExo) than the change in muscle activity (16.3% NoExo), and the effect size is similar for both changes (*d* 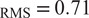 and *d* 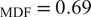 ). The slope of the regression lines was significantly less steep for the quadratus lumborum (*p* = .008) and gluteus maximus (*p* = .045), see Table 2.

**Table 2.** Change in median frequency slope when wearing the Exo with respect to the NoExo condition across the sample (*n* = 14)

	Change in slope (NoExo – Exo)			Paired t-test		
Muscle group	Mean (%MDF/s)	SD (%MDF/s)	Mean (%NoExo)	*t*	*p*-value	Cohen’s *d*
Erector spinae 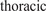	−0.03	0.12	20.1	−0.90	.393	0.28
Erector spinae 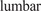	−0.03	0.12	13.6	−1.07	.306	0.29
Quadratus lumborum	−0.05	0.05	10.1	−3.25	.008	0.94
Gluteus maximus	−0.20	0.29	44.0	−2.29	.045	0.69

*Note.* Mean and SD of the absolute change ( 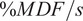 ) and change as % of NoExo condition, as well as the test statistic *t*, the *p*-value, and the effect size (Cohen’s *d*) of the paired samples *t*-test are reported.

The force provided by the textile springs ranged from 45.0 to 278.1 N (mean: 169.1 N; SD: 63.1 N). The force provided by the textile springs was positively correlated with body weight (*r* = 0.64, *p* = .02) and body mass index (*r* = 0.57, *p* = .04) but not with body height or changes in muscle activity.

## 4. Discussion

This work aimed to develop a reproducible, practical, and standardized protocol for measuring fatigue that can be used to benchmark lift- and back-support exoskeleton. We propose to quantify the effect of lift-support exoskeletons on back and hip muscle activity amplitude, as well as the rate of muscle fatigue using a loaded static forward leaning task. The protocol was inspired by previous work conducted by Lamers et al. (2020). To make the protocol suitable for all exoskeleton users, we propose adapting the external load to the users’ body weight and level of fitness. Using this protocol, we were able to induce fatigue in the lumbar erector spinae during the control condition in a sample of participants of both genders, with varying body types and range of fitness. Furthermore, we observed positive effects of the back-support exoskeleton, the Auxivo LiftSuit 2.0, on users’ back and hip muscle load and muscle fatigue.

The level of lower back muscle fatigue induced in the control condition (Lumbar erector spinae; mean: 24.4%, SD: 16.1%) was similar to the fatigue observed by Lamers et al. (2020) in the control conditions (iliocostalis lumborum; mean: 28.5%, SD: 15.5%). We observed significant reductions in muscle fatigue of the quadratus lumborum and gluteus maximus and non-statistically significant reductions at the erector spinae at thoracic and lumbar levels. These findings are similar to the findings of Lamers et al. (2020) who report significant reduction in fatigue of the right iliocostalis lumborum (part of the lumbar erector spinae) and the longissimus thoracis (part of the thoracic erector spinae). In this study, the external load was body weight and fitness dependent (range: 11–21 kg), while in the study conducted by Lamers et al. (2020) the external load was fixed at 16 kg. As a consequence of the fixed external load, Lamers et al. (2020) only report results of 6 out of 12 participants, those being the participants where fatigue was successfully induced. Evidently, both protocols can be used to induce fatigue and show the effect of lift-support exoskeletons. However, we recommend to customize external load to accommodate the inter-subject variability and to enhance statistical power. Our results further indicate that wearing the LiftSuit exoskeleton significantly reduces muscle activity in the back muscles at the level of the thorax. These findings are in line with a previous study evaluating the LiftSuit v1.1 (Goršič et al., 2022), which reports EMG amplitude during a variety of tasks including forward leaning at 30  and 60  . However, in this study, we also observed a significant reduction in EMG amplitude in the erector spinae at lumbar level, as well as in the gluteus maximus, in contrast to the study by Goršič et al. (2022). This might be due to the improvements made in the LiftSuit from v1.1 to v2.0, specifically the introduction of a sizing system to ensure optimal fit and support, and the limitation of the vertical location of the textile springs to prevent incorrect adjustment that can reduce provided support.

A limitation of the study is that based on the data obtained during visit 1 the external load was adapted for six participants, but we failed to verify that the adapted external load reached the fatigue threshold which was also used by Potvin and Norman (1993). In one participant, the reduction in lumbar erector spinae MDF was less than 10% during visit 2 with an external load of 25% of body weight. In this situation, it would be recommended to increase the external load another 5% of body weight. An improvement of the study design would thus be to include a second fatigue measurement with the new external weight. This can be achieved through quick on-site data analysis and repeating the task after a break during visit 1. Another improvement to the study design would be to increase the threshold for increasing the external load from 10% change in MDF to 15% change in MDF. This would make the experiment more taxing but increase the likelihood of observing fatigue during visit 2. To facilitate replication of the protocol and benchmarking efforts in general, a more sparse IMU sensor setup would have been preferred. It is likely that reliable trunk angle feedback can be provided with less kinematic sensors. A further improvement to the study design would be to standardize and increase the break between two measurements. However, randomization of the order of the test conditions mitigates potential effects of remaining fatigue, especially with sufficiently large sample sizes.

Regarding muscle selection, our findings suggest that including the tested lift-support exoskeleton has the potential to reduce muscle activity and fatigue of the back and the gluteus muscles. The high-level rate of fatigue reduction of 44% confirms the importance to include the gluteus muscles. Observed reductions in cardiovascular load while wearing a lift-support exoskeleton, such as observed by Lotz et al. (2009), are likely consequences of reduction in both back and hip muscle activity. Linking changes in cardiovascular load to changes in hip muscle activity requires a longer protocol than the one proposed in this study. Less than 10% of publications on back-support exoskeletons performance reported gluteus activity (De Bock et al., 2022). However, due to the size of the gluteus maximus, and based on our results, we hypothesize that measuring gluteus activity is relevant to understand the systemic effects of lift-support exoskeletons on fatigue development and should therefore be included in a benchmarking protocol.

This study also shows that the effect of a back-support exoskeleton on the development of short-term fatigue in the back and hip muscles can be quantified with three 90 s repetitions of a highly reproducible task. Inducing muscle fatigue through 15 min (Yin et al., 2019) or 45 min (Lotz et al., 2009) of repetitive lifting is a significant effort for both participants and experimenters and is less easy to standardize (i.e., pacing of lifting, maintaining of correct lifting posture while fatiguing). Hence, although inducing fatigue through loaded forward leaning cannot replace studies on lifting, it gives a reliable indication of the level of support provided by the exoskeleton. Since changes in MDF are correlated with both the time a task can be performed (task endurance time; Coorevits et al., 2008) and subjective assessment of fatigue in the lower back (Dedering et al., 1999), the effect of an exoskeleton on this parameter likely translates into relevant changes for users in the field. Therefore, the proposed protocol can in its current form offer significant value when added to existing benchmarking procedures conducted in a laboratory setting. To enhance the value of the results for ergonomists, an adaptation of the protocol for use in quasi-isometric work simulations as well as field studies is of interest (De Bock et al., 2022. Since surface EMG can be measured in the field, the assessment of fatigue during forward leaning work through assessment of the MDF slope is both interesting and feasible.

## 5. Conclusion

This paper proposed a protocol to benchmark exoskeletons and applied the protocol to evaluate the effect of the Auxivo LiftSuit, a commercial passive lift-support exoskeleton, on muscle activity and fatigue at the back and hip during a static forward leaning position. The results show that LiftSuit significantly reduced muscle activity in the erector spinae, both at thoracic and lumbar levels, and the gluteus maximus, and significantly delayed the development of fatigue at the quadratus lumborum and gluteus maximus. Our results show that the effect of lift-support exoskeletons on the development of muscle activity and short-term muscle fatigue can be quantified using the proposed protocol with a reasonable effort from the experimenter and participant. We believe that fatigue measures are important indicators of exoskeleton performance and should be included in standard benchmarking procedures to complement measures of short-term changes in muscle activity amplitude when evaluating the effects of lift-support exoskeletons.

## Data Availability

Key performance indicators can be made available upon request.

## Supplementary material

For supplementary material accompanying this paper visit https://doi.org/10.1017/wtc.2022.32. click here to view supplementary material
